# CT-MR Image Fusion for Post-Implant Dosimetry Analysis in Brain Tumor Seed Implantation- a Preliminary Study

**DOI:** 10.1155/2022/6310262

**Published:** 2022-05-17

**Authors:** Menglong Zhang, Cunkun Chu, Liyin Huang, Bijuan Hu

**Affiliations:** ^1^Department of Minimally Invasive Intervention, Ganzhou People's Hospital, The Affiliated Ganzhou Hospital of Nanchang University, Ganzhou, 341000 Jiangxi Province, China; ^2^Library, Shandong First Medical University & Shandong Academy of Medical Sciences, Tai'an, 271000 Shandong Province, China; ^3^Department of Ultrasonography, Ganzhou People's Hospital, The Affiliated Ganzhou Hospital of Nanchang University, Ganzhou, 341000 Jiangxi Province, China

## Abstract

**Purpose:**

To calculate and evaluate postimplant dosimetry (PID) with CT-MR fusion technique after brain tumor brachytherapy and compare the result with CT-based PID.

**Methods and Materials:**

16 brain tumor patients received MR-guided intervention with Iodine-125 (^125^I) seed implantation entered this preliminary study for PID evaluation. Registration and fusion of CT and MR images of the same patients were performed one day after operation. Seeds identification and targets delineation were carried out on CT, MR, and CT-MR fusion images, each. The number and location of seeds on MR or CT- MR fusion images were compared with those of actually implanted seeds. Clinical target volume (CTV) and dosimetric parameters such as %D90, %V100 and external V100 were measured and calculated. In addition, the correlation of the fusion to CT CTV ratio and other factors were analyzed.

**Results:**

The numbers of fusion seeds were not significantly different compared with reference seeds (t =1.76, p >0.05). The difference between reference seeds numbers and truly extracted MR seeds numbers was statistically significant (t =3.91, p <0.05). All dosimetric parameters showed significant differences between the two techniques (p <0.05). The mean CTV delineated on fusion images was 34.3 ± 33.6, smaller than that on CT images. The mean values of external V100, %V100 and %D90 on fusion images were larger than those on CT images. Correlation analysis showed that the fusion-CT V100 ratio was positively and significantly correlated with the fusion-CT volume ratio.

**Conclusions:**

This preliminary study indicated that CT-MR fusion-based PID exhibited good accuracy for ^125^I brain tumor brachytherapy dosimetry when compared to CT-based PID and merits further research to establish best-outcome protocols.

## 1. Introduction

Radiation treatment in locally advanced head, face and neck cancers engenders a number of adverse effects in this region including radiation necrosis of brain cells and neurocognitive damage [1]. Therefore, a need to optimize radiation protocols assumes very high significance, which in turn necessitates accurate brain dosimetry. Iodine-125 (^125^I) brachytherapy has been applied to brain tumors for almost four decades [2–5]. At present, the permanent implantation of ^125^I seeds remains the preferred technique for tumors in any location within the brain [6]. There have been significant advances in permanent implants for treating brain cancer using LDR Cs-131 brachytherapy by Wernicke and colleagues [7]. Dosimetric analysis after a permanent implant is necessary and could serve as a mandatory procedure designed to guarantee the operation quality, further to revise inadequate implants. The accuracy of postimplant dosimetry (PID) may depend on the definition of the target region and localization of the seeds. Computed tomography (CT) has been the standard imaging modality for PID [8, 9]. Edema often occurs after implantation in brain tumors. Tumors are difficult to distinguish from edema in CT images [10], while the boundary can be found accurately and gross tumor volume (GTV) can be easily outlined in magnetic resonance (MR) images [11]. The major impediment of using MR for PID is that the implanted seeds have no signal on MR image and poor contrast compared with adjacent tissues. On MR images, a seed is characterized by void signal and apt to miss if an anatomic structure around it also appears as void signal such as calcifications, blood vessels, etc. [12] On the contrary seeds are readily identified on CT images by brighter signals.

The fusion of CT and MR images provides the potential for achieving anatomic definition by MR and seed detection by CT at the same time [13]. This method has been used for several anatomical locations in our department. In this study, we used CT-MR fusion technique to calculate PID of brain tumors and evaluate the differences between CT-MR fusion based and CT-based dosimetry.

## 2. Methods and Materials

### 2.1. Patient Population

This retrospective study received approval from our institutional review board. We have utilized MR in guidance of biopsy procedures and ^125^I brachytherapy for brain tumor from 2014 [14, 15]. Sixteen cases, including 2 brain metastases and 14 gliomas, were entered into the study. All 16 patients underwent MR-guided interstitial permanent brain tumor implantation of ^125^I seeds (model No. BT-125-1, Shanghai Sinko Pharmaceutical Co., Ltd) by a specified doctor. Every case received a prescribed dose (PD) of 120Gy with mono-brachytherapy of ^125^I seeds. We summarized reasonable definition of PD from literature [16, 17]. Here PD refers to the pre-set peripheral dose of the target volume in the treatment plan, which is the ‘ideal' minimum dose of the target volume. The pre-treatment planning conforms to double 90% principle (i.e. D90>90%PD and V90>90%PTV, where D90 refers to as the minimum dose received by 90% of the PTV and V90 refers to as the PTV covered by 90% of PD). The number of implanted seeds for every case was recorded for reference seed.

### 2.2. CT and MR Scan Protocols

The mainly interventional suite comprised of a 1.0-Tesla open MRI scanner (Philips Healthcare, Best, Netherlands). Real-time images were viewed by an on-site radiofrequency-shielded liquid crystal monitor in operation room. During operation, the number and position of seeds were confirmed and recorded as criterion to calculate accuracy of post-implanted seed extraction in CT or CT-MR fusion images. T1- or T2-weighted, turbo spin echo MR images with an axial slice thickness/gap of 3 mm/0 mm were acquired using a four-element head array coil (matrix 240×165, field of view 230 × 199) immediately after implantation achieved.

CT scan for PID was completed one day after implantation on a DSCT unit (Somatom Definition, Forchheim, Germany). The images collecting parameters included 0.5-s tube rotation, 16-cm axial volumetric scanning range, 120-kVp tube voltage, 1-mm slice thickness and 0-mm interval. The images were reconstructed into 3-mm slice thickness and an interval of 0-mm, which is same as MR images. Both CT and MR scanning data were stored in the picture archiving and communication system (PACS).

### 2.3. Image Fusion

The ways of correlating CT and MR images are image registration and fusion. The purpose of image fusion is to infiltrate the seed positions highlighted on CT images into MR images. This process was performed using Syngo.via client 3.0 (Siemens) software. The requested data sets (including T1 or T2 MR image and reconstructed CT image data set) were retrieved from PACS via the network and were imported into Singo. These image data sets were input into a common coordinate system. The MR image data set was used as a reference, and CT image data set was reoriented and registered to the MR coordinate system. Some seed locations and anatomic structures which can be distinctly distinguished in both CT images and MR images acted as co-registration marks. By using manual rotation and movement in all the three spatial directions and corrections of a patient position, the same locations of co-registration marks in CT and MR image data sets completely coincided with each other. Once fusion is completed the fusion images were stored to PACS.

### 2.4. Seed Identification

**Figure 1:**
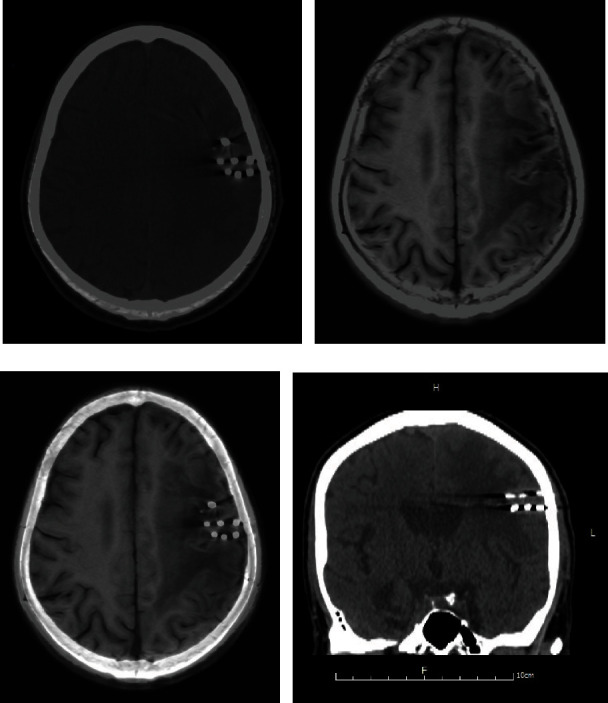
A case to show the same seeds identified on different types of images. (a). CT image; (b). MR T1 FLAIR image; (c). CT-MR fusion image; (d). Reconstructed image along needle path, used to confirm reference seeds.

The number and position of actually implanted seeds for every case were determined as reference seed coordinates by a series of procedures. Specifically, an operator informed the needle path and the number of seeds in each needle. Meanwhile, images along the needle direction were reconstructed with 1-mm slice thickness CT images (Figure 1(d)). The investigator could use suitable window -level and -width of reconstructed images to isolate high-density images and identify seed locations for each patient. Fusion seeds refer to as the seeds extracted from transverse views of CT-MR fusion images. Because fusion images were based on MR images onto which contours of seed position on CT images were “burned”, the fusion seeds appearing in CT-MR fusion images were the same as those on CT images. MR Seeds refer to as the seeds extracted from transverse views of MR images. After the completion of MR seeds statistical analysis, we determined the noise and artifacts by comparing reference seeds, so as to determine the false and missing seeds. All seeds identification (reference seeds, fusion seeds and MR seeds, as shown in Figures 1(a), 1(b), 1(c)) was performed by a specific investigator (MZ), who was calibrated.

### 2.5. Postimplant Dosimetry Verification

For every case, 2 DICOM modalities (MR images and CT-MR fusion images) were downloaded from PACS and transferred to a treatment planning system (TPS, Image Center, Beihang University) for PID verification, which include seed extraction, target contouring and dosimetry analysis, respectively. The interval between processing of the two modalities was more than 1 month to avoid any information of one image that may influence delineation and identification of the other. All delineation and identification were performed by a specific investigator (MZ). Clinical target volume (CTV) was determined by outward adding 5 mm on GTV. Here we defined primary dosimetric parameters such as %D90, %V100, and external V100 in the way of Kristina L. et al. [18] All dosimetry data for CTV was analyzed based on them.

### 2.6. Statistical Analysis

Information about seeds, volume and dosimetry based on CT, MR, and CT-MR fusion images for every case was summed up and analyzed. Data about CTV, external V100, %D90 and %V100 are calculated and listed. Then volumetric or dosimetric ratios of CT-MR fusion images to CT images were estimated. The CT-MR fusion images were compared with CT images for each volume and dosimetric parameters by performing the two-sided paired *t*-test. Correlation analysis for the fusion to CT CTV ratio and other factors were calculated by SPSS 19 statistical software, and p <0.05 was statistically significant.

## 3. Results

### 3.1. Identified Seeds

The numbers of reference seeds ranged from 6 to 57, determined by their recorded number when they were implanted. Seed activities were same as 0.60 mCi at the time they were implanted. The numbers of fusion seeds were not significantly different compared with those of reference seeds (t =1.76, p >0.05). The difference between reference seeds numbers and true seeds numbers extracted on MR images was statistically significant (t =3.91, p <0.05). The differences of seeds numbers among the PID techniques are summarized in Table 1.

### 3.2. Differences in Volume and Dosimetry between CT and Fusion Images

The main target dosimetric parameters, PTV, External V100, %D90 and %V100 were calculated and compared between CT- and fusion- based PID (Table 2). All dosimetric parameters showed significant differences between the two techniques (p <0.05). The mean CTV delineated on CT-MR fusion images was 34.3 ± 33.6, which was smaller than that on CT images. Additionally, the mean values of external V100, %V100 and %D90 on CT-MR fusion images were each greater than those on CT images.

### 3.3. Correlation Analysis

The mean fusion-CT CTV ratio (V_fusion/CT_) was (0.85±0.10). Correlation analysis showed that the fusion-CT V100 ratio was positively and significantly correlated with the fusion-CT volume ratio (r =0.771, p <0.001), and fusion-CT D90 ratio was inversely and significantly correlated with the fusion-CT volume ratio (r =0.697, p =0.003). Total V100 and implanted seed number both showed low and non-significant correlation with the fusion-CT CTV ratio (Table 3).

## 4. Discussion

We have studied the use of CT-MR fusion in post-implant dosimetry in brain tumors. Fusion-based PID was proved to have obvious advantages over CT- or MR- alone based PID. Multiple previous studies addressing fusion-based PID have focused on prostate cancer [19, 20]. Our work is complementary to the application of CT-MR fusion technology in brain tumors.

CT imaging so far remains the best imaging modality for seed identification. Seeds are readily identified on CT images by bright signals. ESTRO/EAU/EORTC recommended that CT imaging was the best way for seed location and extraction [21]. In this study, investigator located and identified fusion seeds on CT or fusion images by using image processing technology without knowing the seeds information in advance. In most cases, the number and location of fusion seeds coincided with those of actually implanted seeds (t =1.76, p >0.05). Therefore, we took CT as the standard method for seeds recognition.

There are two reasons for erroneous identification of seeds on CT. One is Clusters of seeds close together. We designed to implant 1 seed every 1 cm in treatment planning. At the beginning of implantation, the space between adjacent seeds was about 0.5 cm (the seed length along axis is about 0.5 cm). As time passed, the volume of tumor becomes smaller and the seeds become more and more huddled together. Therefore isolating and identifying each seed becomes difficult. The other reason is related with seed coordinates relative to axial images. The standard axial tomography often does not coincide with the seed's long axis, which leads to an object possibly appearing on more than one slice and be regarded as two to three seeds. The images reconstructed along the needle path can display a complete seed on one slice (Figure 1(d)).

On MR images, if the signal intensity and size of the anatomical structure is the same as that of the seed, it is likely to be regarded as a “false seed”. If the seed position is close to the anatomical structure, which appears void signal, it is often thought a “missing seed” [12]. In our study, the seeds correctly identified by MR imaging were lower than those identified by CT imaging. On MR images, gradient echo sequence is more advantageous than T2 weighted sequence for seeds identification [22]. Therefore, we used T2 weighted sequence, T1 weighted sequence, and gradient echo sequence to determine the seed position in the application of MR image for PID.

The advantages of MR imaging include: (i) no ionizing radiation; (ii) excellent soft-tissue contrast [23]. It is an ideal tool to guide seed implantation for brain tumors. In pre-operative treatment planning, the CTV will be defined as tumor plus peripheral edema because the edema is thought to harbor microscopic disease [24]. On the other hand, in post-operative verification, the newly enlarged edematous area is thought to be caused by surgical operation and implanted seeds [25], so it should not be included in the target volume. MR imaging is more accurate than CT imaging in identifying the boundary between tumor and edema [26, 27] and MR-based fusion images are more suitable for TPS.

MR imaging is good at anatomic discrimination but ineffective for seed confirmation. Complementarily, CT imaging can clearly show seeds, but it is not readily able to distinguish the boundary between normal brain tissues and tumors [28, 29]. Therefore, to encompass both high anatomic precision superiority of MR imaging and seed recognition superiority of CT imaging, CT-MR fusion image was utilized to undertake PID. CT-MR image fusion may be the best dose verification method until MR techniques specialized for seed extraction are developed. Recently, 3-dimensional printing non-coplanar template has been used in brachytherapy. Some researchers have suggested it can improve conformity between the preoperative plan and postoperative plan in combination with CT-MR fusion images [30, 31].

**Figure 2:**
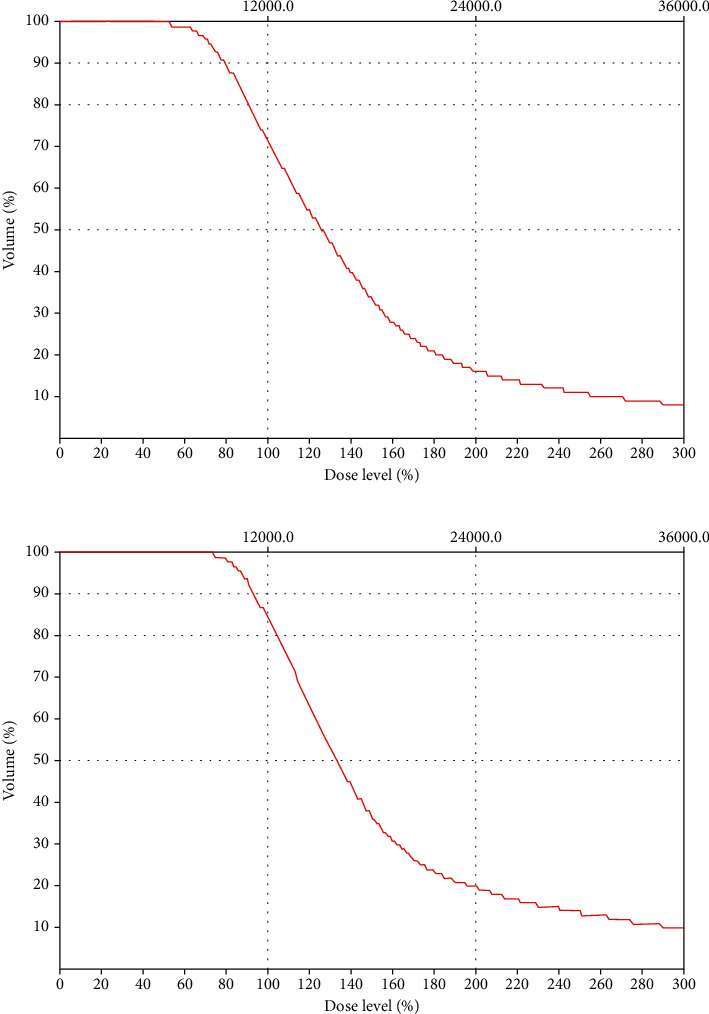
A case to show %V100 and %D90 difference between CT- and fusion-based dosimetry (PD: 12000 cGy). (a). CT-based dose-volume histogram; (b). fusion-based dose-volume histogram. From DVHs, we can find %V100 in CT-based DVH (71.7%) is smaller than that in fusion-based DVH (84.7%). %D90 in CT-based DVH (80.0%) is larger than that in fusion-based DVH (94.2%).

CTVs produced by CT imaging were on average 15% larger than those produced by CT-MR fusion imaging. V100 also supported this fact. Accordingly, %V100 and %D90 on dose-volume histogram produced by CT imaging are different from those produced by CT-MR fusion imaging (Figure 2). Larger CTVs have been proved to be correlated with edema [9, 24]. The fusion CTV excluding edema was smaller than the CT counterpart. Correlation analysis further showed significant relationship between volume ratio (V_fusion/CT_) and fusion/CT dosimetry ratios (V100_fusion/CT_ and D90_Fusion/CT_). Therefore, we can infer fusion-based dosimetry to be superior to CT-based dosimetry. However, CT-MR fusion-based methods are costly and inconvenient because it requires both CT and MR scans. In addition, further research considering the estimation of accuracy of the therapeutic curve range is essential [32] and is an ongoing area of research where novel approaches and algorithms are being trialled [33]. Another consideration is the identification of best MR modality for fusion that yields highest accuracy. While T1 weighted images and CT are considered the best for visualizing interstitial seeds [34], greater research is warranted in this direction. Also, in the present study, the number of I-125 particles was not analyzed and serves as a limitation. Currently, the advances in CT-imaging and post-processing can enable the determination of particle number, spatial arrangement and activity [35]. Here we did not make a detailed study of seed migration. Probabilities of seeds losing and change of seeds position are very small in a short period (only 1 day delay from operation to evaluation) [36, 37]. Therefore, the total number and location of seeds detected during verification can be assumed to be the same as they were just implanted. In addition, the small number of cases in this preliminary study precludes the generalization of these findings and larger studies are essential. Overall these findings are consistent with earlier reports demonstrating the feasibility of CT-MR fusion based dosimetry in brain tumors [38].

## 5. Conclusions

CT-MR fusion-based PID exhibited greater accuracy in ^125^I brain tumor brachytherapy than CT-based PID. Given the advantage of MR-based tissue definition and CT-based seed appearance, further study of CT-MR fusion-based dosimetry is warranted.

## Figures and Tables

**Table 1:** Differences in seed extraction among the PID techniques.

Patient no	Number of reference seeds	Number of fusion seeds	Number of MR seeds		
			True	False	Missing
1	13	13	10	4	3
2	55	61	42	15	13
3	35	36	26	7	9
4	18	18	16	3	2
5	12	12	10	3	2
6	20	21	13	6	7
7	18	17	13	6	5
8	13	13	12	3	1
9	15	15	15	1	0
10	30	31	27	5	3
11	33	36	26	10	7
12	16	16	15	2	1
13	15	16	12	4	3
14	29	28	22	8	7
15	57	53	48	10	9
16	6	6	6	0	0
$\bar{x}\pm s$	24.1 ± 14.9	24.5 ± 15.4	19.6 ± 11.8		
t		-0.848	4.743		
∗p		0.410	<0.001		

∗compared with number of implanted seeds (reference seeds). T2 weighted sequence, T1 weighted sequence, and gradient echo sequence were used to determine the seed position in the application of MR image for PID.

**Table 2:** Volume and dosimetry on CT and fusion images.

Image type	Cases	CTV		External V100		%V100		%D90	
		$\bar{x}\pm s$	Range	$\bar{x}\pm s$	Range	$\bar{x}\pm s$	Range	$\bar{x}\pm s$	Range
CT	16	38.4 ± 35.2	3.70-124	3.73 ± 3.41	0.50-12.3	79.8 ± 13.0	46.1-96.3	86.7 ± 16.4	57.0-114
Fusion		34.3 ± 33.6	2.50-122	4.83 ± 4.68	0.70-17.0	89.2 ± 8.60	60.5-99.0	101 ± 9.4	90.0-122
Ratio fusion/CT		0.85 ± 0.10	0.60-0.98	1.32 ± 0.34	1.02-2.40	1.13 ± 0.12	1.01-1.42	1.19 ± 0.17	1.01-1.65
t		-5.832		3.764		4.566		4.316	
∗p		<0.001		0.002		<0.001		0.001	

∗H_0_: ratio = 1. $\bar{x}\pm s$ = mean±standard deviation; CTV = clinical target volume; external V100 = the volume of the V100 dose cloud outside the target volume; %D90 = the minimum dose received by 90% of the CTV expressed as a percentage of the prescription dose; %V100 = the percentage of the CTV covered by 100% of the prescription dose.

**Table 3:** Correlation analysis of factors associated with the fusion-CT CTV ratio.

	D90_fusion/CT_	V100_fusion/CT_	Total V100	Implanted seed number
$\bar{x}\pm s$	1.19 ± 0.17	0.96 ± 0.04	36.16 ± 35.79	25.44 ± 17.58
Pearson *r*	-0.697	0.771	0.306	0.271
*p*	0.003	<0.001	0.249	0.309
Correlation	Significant	Significant	Low	Low

$\bar{x}\pm s$ = mean±standard deviation; V_fusion/CT_ = ratio of CTV on fusion images to CTV on CT images; V100_fusion/CT_ = ratio of V100 on fusion images to V100 on CT images; Total V100 = total volume of the V100 dose cloud.

## Data Availability

The data used to support the findings of this study are available from the corresponding author upon reasonable request.
